# A New Fault Diagnosis Method for a Diesel Engine Based on an Optimized Vibration Mel Frequency under Multiple Operation Conditions

**DOI:** 10.3390/s19112590

**Published:** 2019-06-06

**Authors:** Haipeng Zhao, Jinjie Zhang, Zhinong Jiang, Donghai Wei, Xudong Zhang, Zhiwei Mao

**Affiliations:** 1 Key Lab of Engine Health Monitoring-Control and Networking of Ministry of Education, Beijing University of Chemical Technology, Beijing 100029, China; 2017400141@mail.buct.edu.cn (H.Z.); zhangjinjie@mail.buct.edu.cn (J.Z.); jiangzn@mail.buct.edu.cn (Z.J.); 2 Beijing Key Laboratory of High-End Mechanical Equipment Health Monitoring and Self-Recovery, Beijing University of Chemical Technology, Beijing 100029, China; 2016200682@mail.buct.edu.cn (D.W.); 2018200649@mail.buct.edu.cn (X.Z.)

**Keywords:** fault diagnosis, diesel engine, Mel frequency cepstrum (MFC), vibrational mode decomposition (VMD), vibration signals

## Abstract

The diesel engine has been a significant component of large-scale mechanical systems for the intelligent manufacturing industry. Because of its complex structure and poor working environment, it has trouble effectively acquiring the representative fault features. Further, fault diagnosis of the diesel engine faces great challenges. This paper presents a new fault diagnosis method for the detection of diesel engine faults under multiple operation conditions instead of conventional methods confined to a single condition. First, an adaptive correlation threshold process is designed as a preprocessing unit to enhance data quality by weakening non-impact region characteristics. Next, a feature extraction method for sound signals based on the Mel frequency cepstrum (MFC) is improved and introduced into the machinery fault diagnosis. Then, the combination of the improved feature and vibrational mode decomposition (VMD) is proposed to incorporate VMD into an effective adaptive decomposition of non-stationary signals to combine it with an excellent feature representation of the vibration signal. Finally, the vector quantization algorithm is adopted to reduce the feature dimensions and generate codebook model bases, which trains the K-Nearest Neighbor classifiers. Five comparative methods were carried out, and the experimental results show that the proposed method offers a good effect of the common valve clearance fault of diesel engines under different conditions.

## 1. Introduction

Diesel engines are the driving force of large-scale mechanical systems widely used in ships and nuclear power plants. Mechanical components of diesel engines will inevitably be prone to break down with high probability because of their severe working environments, featuring problems such as a high speed-variable load, high pressure-high temperature, heavy impact, and serious noise [1,2,3,4,5]. If incipient faults are not discovered in time, they will reduce the reliability and efficiency and eventually cause serious personal safety problems. Therefore, fault detection and diagnosis systems have deservedly attracted an increasing amount of attention, in order to detect potential faults and determine the fault severity so the situation’s further deterioration can be prevented [6,7,8]. Compared with the acoustic signal and thermal parameters, the vibration signal often contains significant dynamic information about mechanical parts, such as the piston-connecting rod assembly reciprocation, crankshaft rotation, and valve train opening and closing. In addition, the signal acquisition is simple and non-embedded, so vibration signal analysis has been an alternative, effective data-driven method [9,10,11,12].

Great progress has been made in mechanical fault diagnosis, but some challenges still remain to be addressed for real practical applications, such as fault feature extraction under multiple operation conditions, which determines the efficiency and effectiveness of detection and diagnosis based on vibration signals to a large extent [13]. There exist three intrinsic drawbacks: (1) The vibration signals acquired from actual sites are generally nonlinear and nonstationary under multiple operation conditions; (2) the valuable fault features are often contaminated by some uncorrelated components, such as serious noise; (3) although multiple hierarchical nonlinear transformation can automatically learn features from the original data, it is necessary for deep learning to tune enough hyper parameters with sufficient normal-fault data and also use expensive computing tools. Consequently, some advanced feature extraction techniques for machinery systems are expected to be developed. Traditional artificial features are mainly extracted by statistical methods performed in various space domains [14,15,16,17,18]. Time-domain methods are only applicable to stationary and linear signals. Although frequency-domain or time-frequency domain methods are considered as the methods appropriate for non-stationary signals, they have difficulty in selecting basic functions and require significant experience. Unlike the fix function decomposition methods, the biggest advantage of the adaptive decomposition methods is that there is no need to set any basis function in advance, such as empirical mode decomposition (EMD), local mean decomposition (LMD), or ensemble empirical mode decomposition (EEMD), which can decompose non-stationary signals based on itself [19,20,21]. Despite freeing the system from its basic functions, the above methods still present some limitations, such as mode mixing and an end-point effect in the EMD method, as well as parameter selections of white noise in the EEMD method. As an optimal variant of EEMD, an iterative non-recursion adaptive method called variational mode decomposition (VMD) was invented to decompose multiple component nonstationary signals by Dragomiretskiy and Zosso in [22]. Here, intrinsic mode functions (IMFs) are acquired adaptively from the multiple component nonstationary signals with the disadvantage of no end-point effect, no mode mixing, and good adaptability. Cai et al. used the VMD method for compound fault detection of gear cracks and rollers [23]. Huang et al. applied the VMD method for the fault diagnosis of rolling bearings on a high speed railway [24]. Mao et al. proposed a novel decomposition rule based on variational mode decomposition for conrod small end bearing knock fault diagnosis [25]. It is no surprise that variational mode decomposition has achieved satisfying results for a variety of applications. Nevertheless, it is still at a very early stage for reciprocating machinery systems. Furthermore, vibration signals are always disturbed by different conditions and strong noise. In addition, the existing research has primarily treated the variational mode decomposition method as a tool to reduce strong noise, which neglected implicit feature information in each component. Thus, the study of a new analysis method for each component has been the most pressing task.

Maintenance Technicians (Ma Tec) have an excellent ability to achieve a “one-listen” of the operation status of system through binaural means. The reason is that the human acoustical system is a highly intelligent sound recognition system, so an approach to generate fault features of multiple operation conditions is referenced to analyze the probability of the Mel frequency cepstrum (MFC) for mechanical fault diagnosis based on each component of the variational mode decomposition. Balsamo et al. introduced an adapted MFCC as a feature and used the Mahalanobis distance to calculate these features for damage detection of structural health monitoring [26]. Combining the independent component analysis with a radial basis function network, Ramuhalli et al. used MFCC to remove the noise of of concrete bridge decks and overcome the subjectivity of the inspector [27]. It can be seen that MFCC features generated by MFC have drawn the attention of numerous researchers in the field of structural health monitoring (SHM). Thus, it would also be a good and innovative way to introduce MFCC features into the fault detection and diagnosis of diesel engines. However, as a simple feature limited to noise-free sound systems, it is evident that MFCC will not meet the requirements for the fault detection and diagnosis of diesel engine [28]. More worrying is that the raw vibration signal collected from sensors usually contains a large amount of invalid data due to high frequency acquisition, which may contaminate impact features and increase computational burden. To solve the issues described above, an adaptive correlation threshold method is developed as a data pre-processor to weaken the invalid data, and then an improved VMD-MFCC feature (IVMD-MFCC) is proposed, which incorporates VMD into the effective adaptive decomposition of non-stationary signals to combine it with the excellent feature representation of the vibration signal. A fault detection and diagnosis system generally consists of signal acquisition, feature extraction, dimension reduction, and pattern recognition. Once the relevant features have been extracted from the acquired signals through signal analysis methods, the next steps are dimension reduction and pattern recognition. Most popular dimension reductions include linear discriminative analysis (LDA), principal component analysis (PCA), and isometric feature mapping (ISOMap) [29,30,31]. However, these methods are used to select principal components and remove redundant information, which inevitably results in some useful data loss. Comparatively, vector quantization (VQ) finds a set of a parts-based representation of the original data or feature vectors, which can compress data and retain original information to the greatest extent simultaneously, so it is widely applied in signal encoding and image synthesis [32]. As far as we know, no papers have reported on the VQ of mechanical fault detection and diagnosis, especially the reciprocating machinery. As a result, by using the compressed features as the input of the K-nearest neighbor (KNN), a new fault detection and diagnosis method has been investigated for the application of a diesel engine under multiple operation conditions based on IVMD-MFCC. Finally, the efficiency of the proposed method is confirmed by practical application to the fault diagnosis of three common diesel engine problems, including small valve clearance faults and large valve clearance faults. The first problem is solely the inlet valve faults. The second problem is solely the exhaust valve faults. The third problem is concurrently the inlet and exhaust valve faults of a single cylinder. The results confirm that the proposed method is more efficient compared with other methods. The main innovations of this paper are as follows:

1. In order to overcome the interference of complex operation conditions, this paper presents a novel representative feature from vibration signals by incorporating VMD into the effective adaptive decomposition of non-stationary signals to combine it with the excellent feature representation of MFCC.
2. In order to wipe out the strong noise and enhance signal improvement, an adaptive correlation threshold method is first proposed to weaken the invalid data before the feature extraction.

The rest is organized as follows. The diesel engine testbed and signal analysis are described in Section 2. Methodologies based on the improved MFCC are developed in Section 3. The comprehensive procedure of the proposed method is presented in Section 4. Diagnosis results of valve clearance fault are presented in Section 5. Lastly, the conclusions are described in Section 6.

## 2. Diesel Engine Test-Bed and Signal Analysis

### 2.1. Diesel Engine Equipment and Data Acquisition

**Figure 1:**
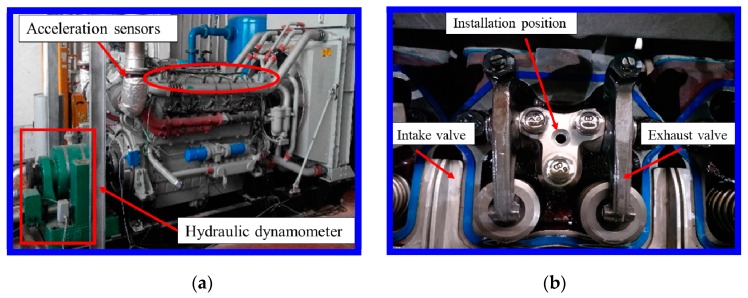
Diesel engine and valve: (**a**) V-type 12 cylinder diesel engine and sensors; (**b**) installation position and intake and exhaust valve.

The diesel engine TBD234V12, as a research object, is a V-type, four-stroke, direct injection marine diesel engine. The corresponding technical parameters are that cylinder bore and cylinder stroke are 128 mm and 135 mm, respectively, with a compression ratio of 15:1, and the consecution power/speed is 373/1500 kW/rpm. The load is regulated by a hydraulic dynamometer connected to a diesel engine through an elastic coupling. Vibration signals are collected by accelerometers installed on cylinder heads. The sample frequency is set as 51.2 kHz, and each sample length is intercepted at 4096. Pictures of the experimental device, and its intake and exhaust valves, are presented in Figure 1.

### 2.2. Establishment of Diesel Engine Valve Fault

The valve is very essential to the performance of a diesel engine to control air suction and the exhaust of the combustion chamber, which consists of an intake valve and an exhaust valve. Nevertheless, its structures has a high probability of failures, such as valve wear, due to its working environment featuring high temperature, high pressure, and intense impact, which leads to improper valve timing, thereby reducing diesel engine performance. In consideration of thermal expansion, the normal valve clearance (NVC) should be a constant, of which the intake valve and exhaust valve is 0.3 mm and 0.5 mm, respectively. Six typical valve clearance faults are summarized based on the diesel engine maintenance records of TBD234V12. Table 1 shows detailed situations of valve clearance.

**Figure 2:**
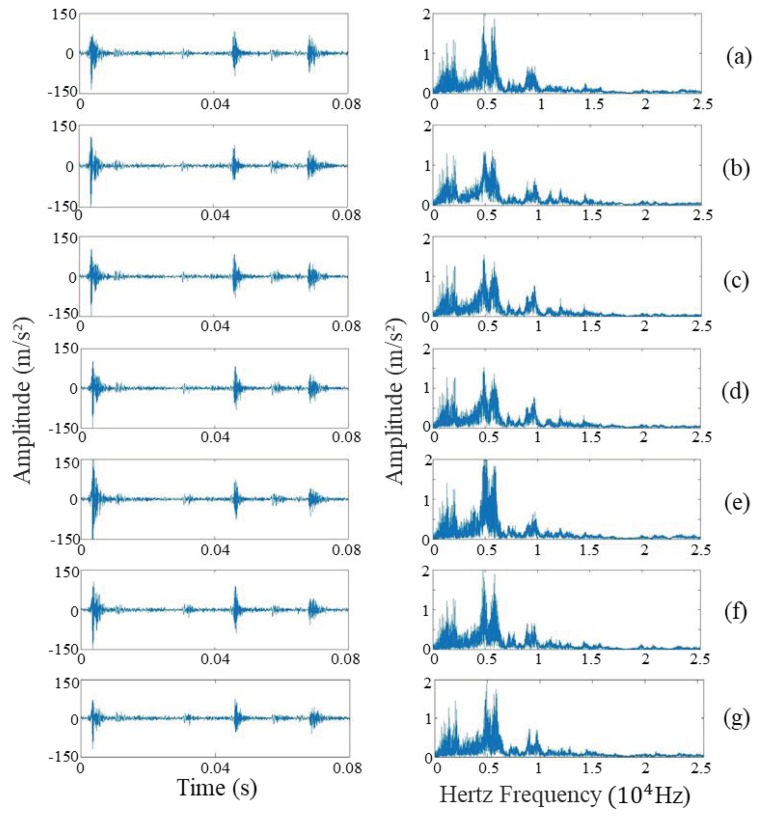
Original vibration signals of seven valve clearance states and corresponding frequency spectrums: (**a**) NVC, (**b**) SFI, (**c**) LFI, (**d**) SFE, (**e**) LFE, (**f**) SFIE, (**g**) LFIE.

The multiple operation conditions are simulated under twelve operating conditions in Table 2. In this experiment, the dataset is made up of twelve subsets under twelve operating conditions, and a total of 1100 integer cycle vibration signals are collected in each valve state. The 120 samples and other 980 samples are stochastically assigned as training samples and testing samples, severally. The calculations are run in an Intel^®^ Xeon^®^ Gold 6142 workstation with 128 GB RAM. The original vibration signals of the seven valve clearance states and corresponding frequency spectrums are depicted in Figure 2.

### 2.3. Time-Frequency of Sound and Vibration Signals 

**Figure 3:**
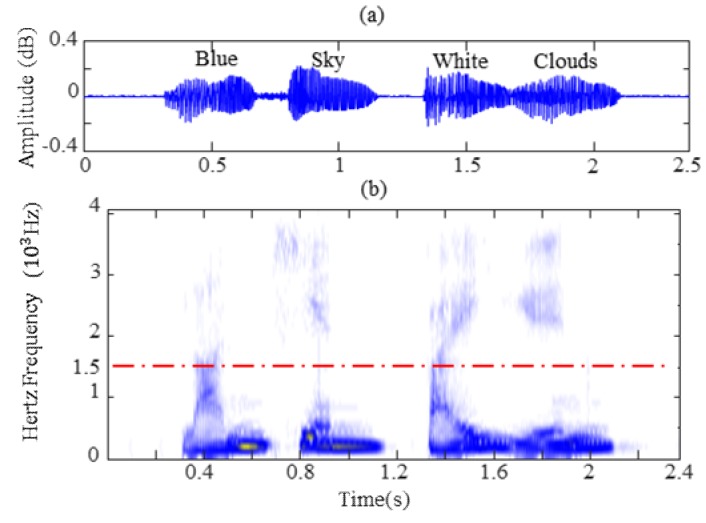
Sound signal: (**a**) original signal waveform; (**b**) STFT.

**Figure 4:**
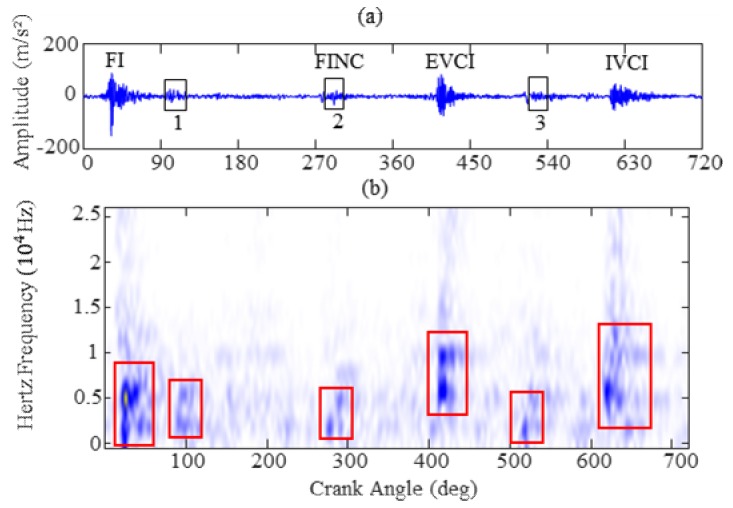
Vibration signal of NVC: (**a**) original signal waveform; (**b**) short time Fourier transform spectrum (STFT).

**Figure 5:**
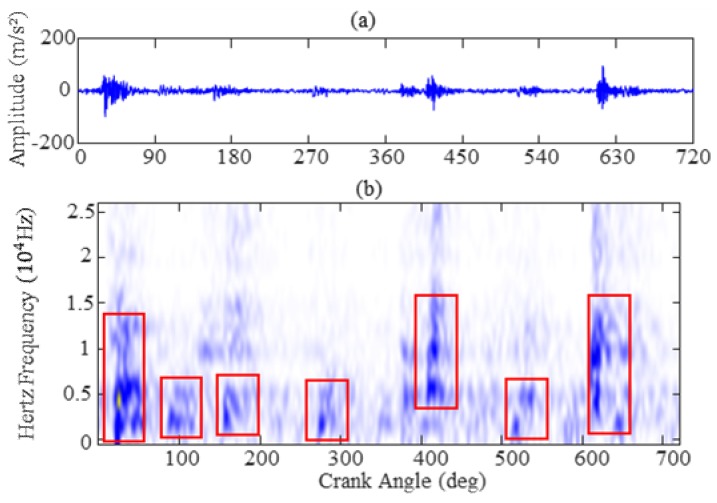
Vibration signal of SFI: (**a**) original signal waveform; (**b**) STFT.

In short, speech recognition is a process that depends primarily on frequency components for phoneme analysis. Figure 3 shows the original signal waveforms and the short time Fourier transform (STFT) spectrum of four sound words. The sound signals were obtained from literature [33]. It is observed from Figure 3a that the pronunciation of each word generates a signal impulse in the original signal waveforms. In Figure 3b, each sound word has different frequency distributions for four sound words whereas the main frequency components are mainly concentrated on the low frequency and close to 1.5 kHz (red imaginary line in Figure 3b). In order to illustrate the similar frequency distribution characteristics and verify the feasibility of extracting features from the vibration signal based on MFC, the time-frequency distribution of the vibration signal from different valve clearance states is also studied in this section. The original signal waveforms and short time Fourier transform spectrum (STFT) images are presented in Figure 4 and Figure 5, corresponding to vibration signals of normal valve clearance (NVC) and small clearance fault of intake valve (SFI), respectively. From Figure 4a and Figure 5a, it is obvious that both vibration signals and sound signals have some similar impulse characteristics, but the difference is that they have different physical meanings, as the sound signal corresponds to a word pronunciation and the vibration signal corresponds to the impact of a specific state, such as a valve opening, a valve closing, or fire combustion. Similarly, it can be seen from Figure 4b and Figure 5b that the STFT spectrums of vibration signals of NVC and SFI have also a certain frequency range, in which the frequency range of the NVC state is about 0–10 kHz, and frequency range of the SFI state is about 0–15 kHz. In addition, the sound signal and vibration signal have a common feature in the frequency range, that is, there are more frequency components in the low frequency range and fewer frequency components in the high frequency range, which conforms to the Mel frequency distribution. In general, it is obvious that the vibration signal of diesel engine is highly similar to the sound signal, which is a good choice for constructing the feature representation methods of the vibration signal similar to the “acoustical system” based on the Mel scale principle.

The FI refers to the fire impact of the sensor-mounted cylinder; FINC is used to indicate the fire impact of adjacent cylinders; IVCI and EVCI is referred to the closing impact of intake and exhaust valve respectively.

## 3. Methodology Based on Improved MFCC

### 3.1. Mel Frequency Cepstrum Coefficient

**Figure 6:**
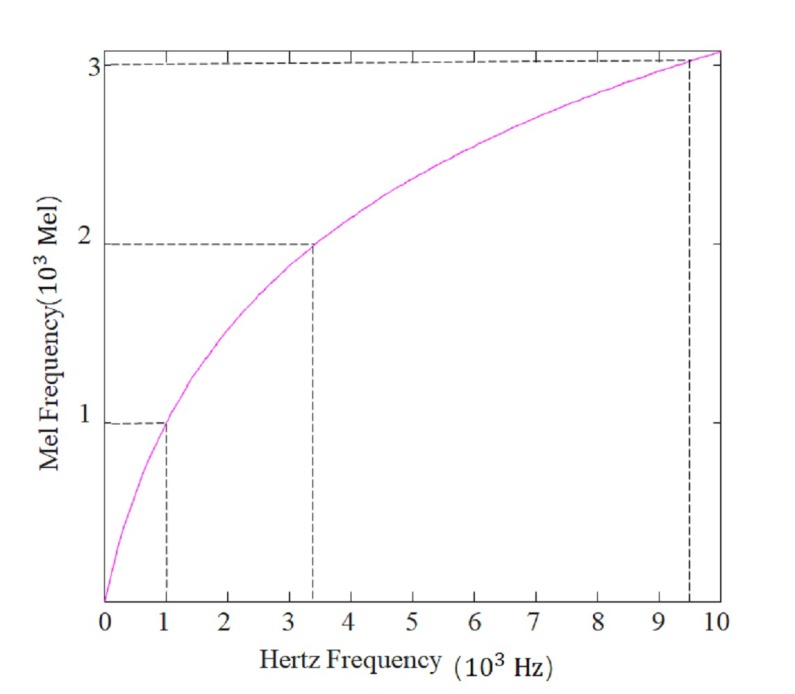
Transformation between the Hertz and Mel frequencies.

The early papers about cepstrum analysis were primarily used to analyze seismic or communication signals. The cepstrum has been widely used in the fault detection of rotating machinery [34,35]. However, little research has been done on cepstrum for reciprocating machinery fault diagnosis, in which frequency domain analysis methods were considered to be less effective. MFC is also a special cepstrum analysis, whose original idea is to imitate how human being perceive their own acoustical system. MFC distributes more weight to lower frequencies while traditional cepstrum analysis distributes equal weight. The situation is similar to frequency distribution in fault detection and diagnosis since the information covered in the lower frequency range is generally more valuable in the higher frequency. It has come to light that human ear perception frequency is 20–20,000 Hz. In 1937, Volkmann and Newman [36], through experimentation, reached the conclusion that there existed a non-linear relationship above 1000 Hz between the Mel frequency and Hertz frequency. So far, there is no sole transformation formula, but a formula that is widely used is shown as Equation (1). The corresponding diagram is presented in Figure 6:

(1) $$Mel(f)=2595\log10(1+\frac{f}{700})$$

where *f* is Hertz frequency and *Mel*(*f*) is Mel frequency.

**Figure 7:**
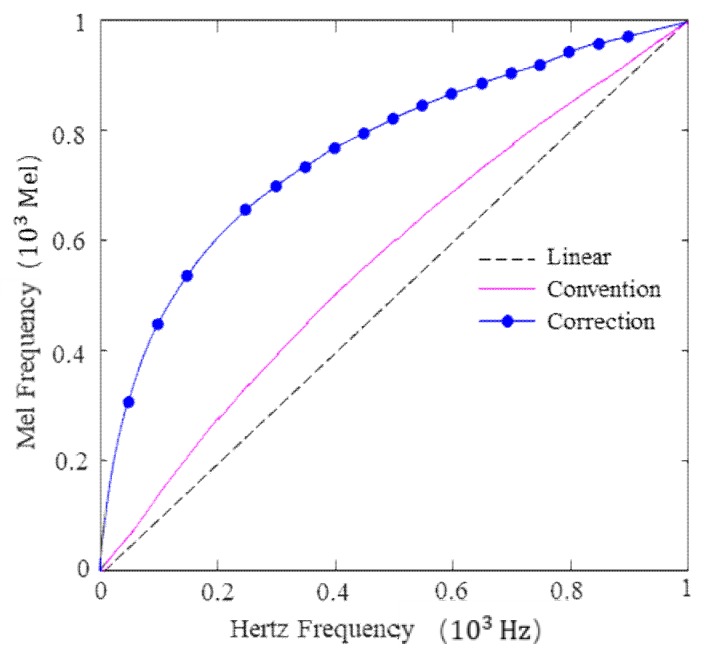
Local Transformation between the Hertz and Mel frequencies.

Unfortunately, as shown by the imaginary and solid lines in Figure 7, it is observed that the conventional transformation curve is very close to a linear relationship between 0 and 1000 Hz, which is mainly contributed to the focus on the frequency above 1000 Hz omitting frequencies below 1000 Hz, in order to imitate the acoustical system [37]. Considering the fact that a frequency below 1000 Hz is still linearly relevant (as per Equation (1)), we have made a supplement to the original formula to mimic the trends above 1000 Hz, as shown in Equation (2). A corresponding relationship of less than 1000 Hz is shown by the dash dotted line in Figure 7:

(2) $$Mel(f)=175\log2(1+\frac{f}{20})\;0\leq f<1000.$$

### 3.2. Variational Mode Decomposition

VMD belongs to a novel adaptive decomposition technology, which can decompose non-stationary signals into *k* mode components. Basic procedures are considered as the establishment and solution of a constrained variational model. The goal of the resulting constrained variational model is to minimize the summation of the estimated bandwidth for all mode components using the Hilbert transform, exponential tune, and Gaussian smooth, e.g. the squared *L*^2^-norm. The constrained variational model is shown as follows:

(3) $$\begin{array}{l} \underset{\left\{u_{k},\omega_{k}\right\}}{\min}\left\{{\sum_{k}\left\|\partial_{t}\left[\left(\delta (t)+\frac{j}{\pi t}\right)*u_{k}(t)\right]e^{-j\omega_{k}t}\right\|}_{2}^{2}\right\}\\ s.t.\sum_{k}u_{k}=x(t) \end{array}$$

A castigatory quadratic *α* and Lagrangian multiplicator operator *λ*(*t*) are integrated into Equation (3), of which the former ensures a nice convergence property and the latter provides constrained strict enforcement. Hence, after converting the constrained problem into an unconstrained problem, the extended Lagrangian function ℒ is described in Equation (4):

(4) $$\begin{array}{r} ℒ\left(\{u_{k}\},\left\{\omega_{k}\right\},\lambda \right)=\alpha {\sum_{k}\left\|\partial_{t}\left[\left(\delta (t)+\frac{j}{\pi t}\right)*u_{k}(t)\right]e^{-j\omega_{k}t}\right\|}_{2}^{2}\\ +{\left\|f(t)-\sum_{k}u_{k}(t)\right\|}_{2}^{2}+\langle \lambda (t),f(t)-\sum_{k}u_{k}(t)\rangle \end{array}$$

where *t* denotes time, *δ*(*t*) is the Dirac distribution function, *u_k_* denotes the decomposed *k* mode components, *j* is an imaginary unit, *ω_k_* is defined as the central frequency, and *x*(*t*) is the original signal.

We use the alternate direction of multiplicators (ADMM) to continually update each mode component and central frequency. In this way, the saddle point of the extended Lagrangian function is obtained. The *L*^2^-norm Parseval/Plancherel Fourier equidistance can decompose the optimal solution in time-domain into a series of iterative sub-optimal problem in frequency-domain. The quadratic optimization of mode component, central frequency and Lagrangian multiplicator can be expressed in Equations (4)–(6):

(5) $${\overset{⌢}{u}}_{k}^{n+1}(\omega )=\frac{\overset{⌢}{f}(\omega )-\sum_{i\neq k}{\overset{⌢}{u}}_{i}(\omega )+\frac{\overset{⌢}{\lambda }(\omega )}{2}}{1+2\alpha (\omega -\omega_{k}{)}^{2}}$$

(6) $$\omega_{k}^{n+1}=\frac{\int_{0}^{\infty }\omega {\left|{\overset{⌢}{u}}_{k}(\omega )\right|}^{2}d\omega }{1+2\alpha (\omega -\omega_{k}{)}^{2}}$$

(7) $${\overset{⌢}{\lambda }}^{n+1}(\omega )={\overset{⌢}{\lambda }}^{n}(\omega )+\tau \left(\overset{⌢}{f}(\omega )-\sum_{k}{\overset{⌢}{u}}_{k}^{n+1}(\omega )\right)$$

In Equations (4)–(6), ${\overset{⌢}{u}}_{k}^{n+1}(\omega )$, $\omega_{k}^{n+1}$ and ${\overset{⌢}{\lambda }}^{n+1}(\omega )$ are the corresponding Fourier transformations. *τ* is a noise tolerance parameter, and the default parameter is 0.

Finally, each mode component can be obtained until the convergence criteria are satisfied or the number of iterations, namely *n*, are equal to *N*. γ is set to 1*e*^−6^, and the formula is presented in Equation (8):

(8) $$\sum_{k}\frac{{\overset{⌢}{u}}_{k}^{n+1}(\omega )-{\overset{⌢}{u}}_{k}^{n}(\omega )}{\left\|{\overset{⌢}{u}}_{k}^{n}\right\|}<\gamma .$$

## 4. Comprehensive Procedure of the Proposed Method

As mentioned in Section 1, the advantages of VMD and MFC are combined in a complementary way that uses VMD for decomposing non-stationary signals and MFC to extract features from the mode components above, as acquired by VMD. However, under the influence of strong noise and high frequency collection, the signal impact features are not obvious, and its proportion is low, so it is not applicable to directly introduce VMD and MFC into the fault detection and diagnosis of a diesel engine. Therefore, an adaptive correlation threshold method is first proposed to weaken the invalid data before the feature extraction.

### 4.1. Signal Improvement

The correlation coefficient is a general statistical indicator used in signal processing, which can quantitatively characterize signal similarity. To calculate the correlation coefficient, the sliding window is used to traverse through the integer cycle signal, and the adaptive threshold is applied to locate the impact features. The equations are as follows:

(9) $$R_{a}=\sum_{m=1}^{N_{wl}}x_{a-1}(m)x_{a}(m+N_{ss})$$

(10) $$y(t)=\left\{ \begin{array}{l} x(t),R_{a}>R_{rms}\\ \theta ,\mathrm{otherwise} \end{array}\right.$$

(11) $$\begin{array}{l} R_{rms}=\kappa \sqrt{\frac{1}{M}\sum_{i=2}^{M}Ra}\\ M=(L-N_{wl})/N_{ss}+1 \end{array}$$

where *Ra* is the correlation coefficient between the (*a* − 1)th and *a*th sliding window (*a* = 2,3,…,*M*). *N_wl_* represents the length of sliding window (default *N_wl_* = 256) and *N_ss_* is the moving step size of the sliding window (default *N_ss_* = 100). *x*(*t*) is the raw signal, while *y*(*t*) is the processed signal via signal improvement of the adaptive correlation threshold. *θ* is the substitute valve of non-impact data (default $\theta =10^{-4}$). *R_rms_* is the mean square root of the correlation coefficient of the integer cycle signal. *M* is the sliding window number, and *L* is the integer cycle signal length.

**Figure 8:**
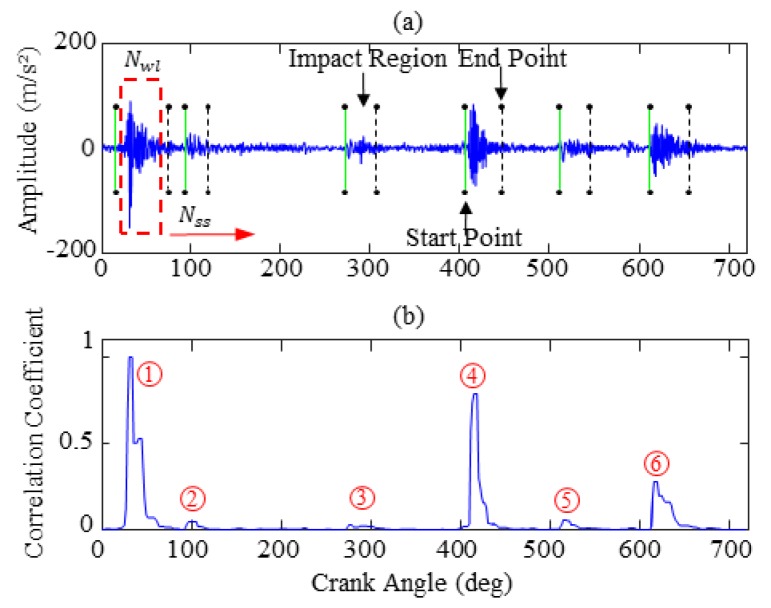
Adaptive correlation threshold processing: (**a**) impact location; (**b**) correlation coefficient.

In Equation (11), κ is a scale parameter, which can control the proportion of the impact and non-impact data. If the κ value is too large, it will hide the “true impact” and leave out some useful information. Nevertheless, a lower value may generate a “false impact” and lead to frequent false alarm. In this experiment, the scale parameter κ is finally adjusted to 0.5 and can successfully capture all impact characteristics of the raw vibration signal. As shown in Figure 8, the results of an adaptive correlation threshold processing are obtained. These results include the impact location and correlation coefficient calculation. In Figure 8a, the red box stands for the sliding window with the length *N_wl_* and moving step *N_ss_*, solid line and dotted lines represents the impact start point and the impact end points respectively, of which the middle part is the impact region. It is observed from Figure 8 that through the adaptive correlation threshold, the six impacts of the raw vibration signal are completely captured, which is beneficial for improvement of the original vibration signal.

**Figure 9:**
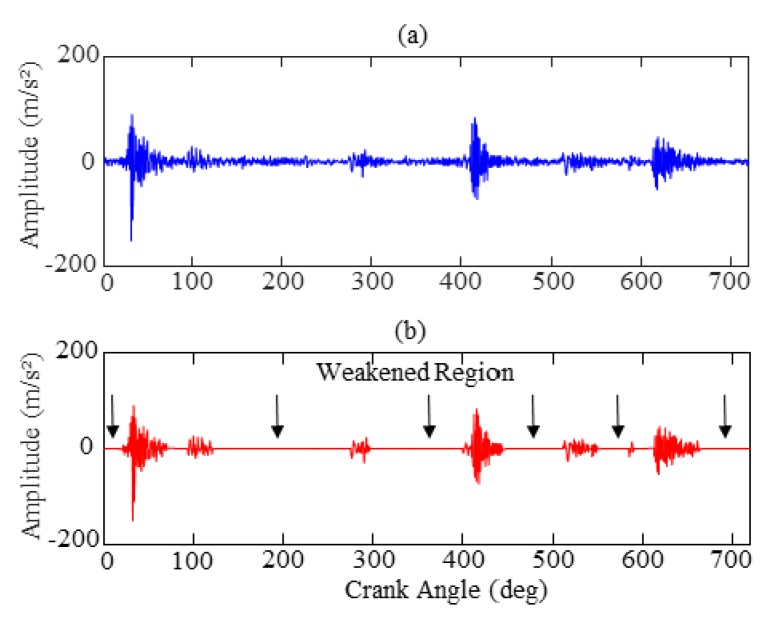
Comparison results of preprocessing: (**a**) original vibration signal; (**b**) processed signal.

Considering the influence of high frequency collection and strong noise, the original vibration signal is selectively weakened to strengthen the impact characteristics through Equation (10). Figure 9 exhibits the effectiveness of this method, in which the top portion is the raw vibration signal and the bottom portion is the processed signal after using an adaptive correlation threshold. As can be seen from the figures (where the arrows are pointing), it is apparent that the processed vibration signal has significantly weakened the invalid data region, which highlights the impact characteristics.

### 4.2. IVMD-MFCC

Based on the principle of VMD and MFC, the detailed descriptions of feature extraction are as follows:

- **Step 1:** Input the raw signal *x*(*t*) and obtain a processed signal *y*(*t*) by utilizing the adaptive correlation threshold.
- **Step 2:** Initialize the parameters $\left\{u_{k}^{1}\right\}$, $\left\{\omega_{k}^{1}\right\}$, $\lambda^{1}$, *n* and step-wise decomposition by using VMD to obtain mode components $\left\{u_{k}\right\}=\left\{u_{1},u_{2},\ldots ,u_{k}\right\}$ (default *k* = 3).
- **Step 3:** Preprocess mode components $\left\{u_{k}\right\}$. To compensate for high frequency loss, each mode component is first weighed, and the weighted filter is shown in Equation (12), where *ρ* represents a constant (default *ρ* = 0.96):
  (12) $$H(z)=1-\rho z^{-1}.$$
  Then, a framing and hamming window are used successively to convert non-stationary signals into quasi-stationary signals, thereby reducing frequency leakage.
- **Step 4:** To carry out fast a Fourier transform (FFT). The FFT transformation is presented in Equation (13), where *p* represents the *p*th line in frequency-domain, *N* is the data point number of $u_{k}^{l}(s)$, and *l* refers to the *l*th sub-frame signal:
  (13) $$Y(l,p)=\sum_{s=0}^{N-1}u_{k}^{l}(s)e^{-p\frac{2\pi }{N}}.$$
- **Step 5:** To calculate the linear spectrum energy of each frame signal. Linear spectrum energy corresponding to the *l*th sub-frame signal is represented in Equation (14):
  (14) $$E(l,p)={\left[Y(l,p)\right]}^{2}.$$
- **Step 6:** Design a series of triangular filters named Mel filter banks, calculate the Mel-frequency spectrum energy of each frame signal, and then take the logarithm:
  (15) $$\mathrm{lg}[S(l,p)]=\mathrm{lg}[\sum_{p=0}^{N-1}E(l,p)H_{fr}(p)]\;0\leq fr\leq F$$
- **Step 7:** To introduce discrete cosine transform (DCT) and extract the IVMD-MFCC features. This relationship is expressed as Equation (16):
  (16) $$IM=\sqrt{\frac{2}{F}\sum_{p=0}^{F-1}\mathrm{lg}[S(l,p)]\cos[\frac{\pi n(2p-1)}{2F}]}$$
  where *IM* stands for the IVMD-MFCC feature of *l*th sub-frame signal, *S*(*l*, *p*) represents the Mel-frequency spectrum energy, and *F* is the number of Mel filters or features (*fr* = 0,1,…,*F*), (default *F* = 20).

**Figure 10:**
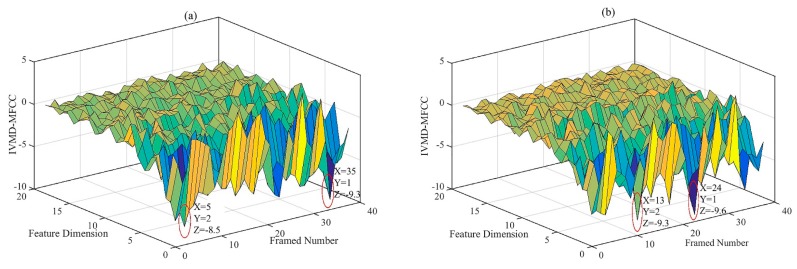
Results for a new feature IVMD-MFCC: (**a**) NVC; (**b**) SFI.

**Figure 11:**
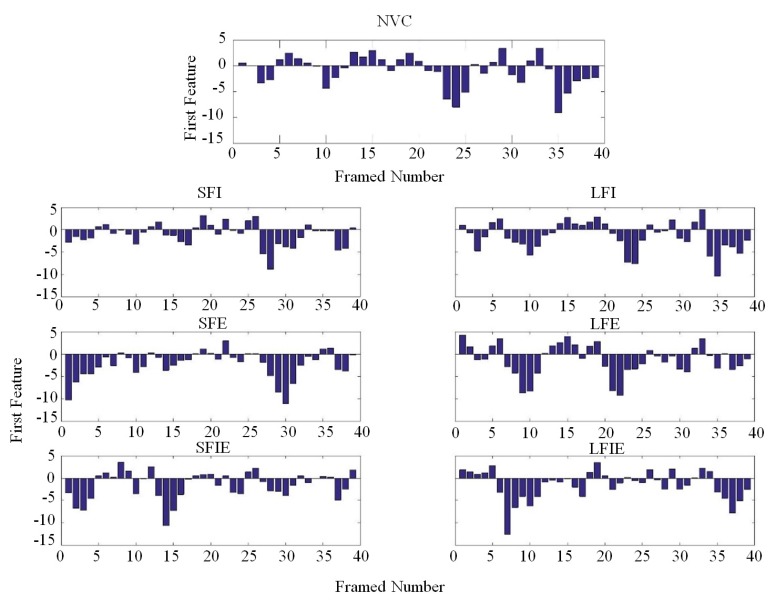
First feature diagrams of the improved VMD-MFCC (IVMD-MFCC) with various states.

In order to better understand the new feature IVMD-MFCCs, Figure 10 shows the new features of two states (NVC and SFI) after being extracted and represented by the proposed method from a certain mode component. The framed number stands for the number of segments of the vibration signal by sliding window, the feature dimension stands for the new feature number of IVMD-MFCC, and the vertical axis stands for the value of IVMD-MFCC. Note that since the IVMD-MFCC with a higher dimension is close to 0, only features of the first 19 dimensions are selected as the input for the purpose of improving the efficiency in this paper. Figure 10a shows detailed information for the IVMD-MFCC of the NVC state, of which the maximum absolute value occurs in the 35th framed vibration signal and the sub-maximum absolute value is located at the 5th framed vibration signal. However, for the SFI state in Figure 10b, the maximum and sub-maximum absolute value occurs in the 24th and 5th framed vibration signals, respectively. It can be seen that IVMD-MFCC changes with the diesel engine valve clearance state. Furthermore, the characteristic differences of NVC and SFI are mainly reflected in the first-dimension feature. Figure 11 shows that the first-dimension feature diagrams of IVMD-MFCC with various faults. The variations of first dimension features between the normal state and various fault states are easy to determine from the figures.

### 4.3. Vector Quantization

The IVMD-MFCC features belong to high-dimensional feature sets, which will undoubtedly increase the model calculation and complexity. To address this difficulty, a novel dimension reduction method called VQ is proposed in this section. It is noticed that the codebook generated by VQ can dramatically reduce the feature dimensions, which removes redundant information and strong disturbances.

The basic principle of VQ is to separate feature vectors into some subspaces and find the corresponding vector representation. The codebook directly determines the signal compression performance of VQ. In this paper, the Linde-Buzo-Gray (LBG) algorithm is used to design the codebook. The principle is simplified, as shown in Equation (17):

(17) $$Y_{c}=Q(X_{ii})\;1\leq c\leq J,1\leq ii\leq F.$$

Supposing that the training set is $X=\left\{x_{1},x_{2},\ldots ,x_{F}\right\}$, the initial codebook is $Y^{\left(0\right)}=\left[y_{1}^{\left(0\right)},y_{2}^{\left(0\right)},\ldots ,y_{c}^{\left(0\right)}\right]$, and λ represents the distortion threshold (initial value is an infinite number). According to the principle of the nearest neighbor condition, spatially partitioning the *J* subspaces of training set:

(18) $$X:\to SU:\left[SU_{1}^{\left(n\right)},SU_{2}^{\left(n\right)},\ldots ,SU_{c}^{\left(n\right)}\right].$$

To calculate the total distortion of all subspaces, the equations are shown in Equation (19) and Equation (20), where $d\left(X,Y_{c}^{\left(n-1\right)}\right)$ represents the Euclidean distance between the training set *X* and codebook *Y_c_* at *n* iteration.

(19) $$d\left(X,Y_{c}^{\left(n-1\right)}\right)=\frac{1}{F}\sum_{ii=1}^{F}{\left(x_{ii}-y_{c}\right)}^{2}$$

(20) $$D^{\left(n\right)}=\sum_{c=1}^{J}\sum_{x\in SU_{c}^{n}}d\left(X,Y_{c}^{\left(n-1\right)}\right)$$

To calculate the relative error of total distortion between two adjacent iterations and find a new codebook vector, if Equation (21) is satisfied, then stop the iteration and $Y^{\left(n\right)}=\left[Y_{1}^{\left(n\right)},Y_{2}^{\left(n\right)},\ldots ,Y_{c}^{\left(n\right)}\right]$ is considered to be the new codebook vector, otherwise return to Equation (18):

(21) $$\frac{D^{\left(n-1\right)}-D^{\left(n\right)}}{D^{\left(n\right)}}\leq \psi .$$

### 4.4. K-Nearest Neighbor (KNN)

KNN is a machine learning method to calculate the distance between a testing sample and K training samples rather than a single training sample, which has a good time efficiency and identification accuracy. Suppose the training sample set is $T_{ts}=\left\{T_{1},T_{2},\ldots ,T_{TS}\right\}$, the label set is $W_{ls}=\left\{W_{1},W_{2},\ldots ,W_{LS}\right\}$, and the testing sample set is $S_{ss}=\left\{S_{1},S_{2},\ldots ,S_{SS}\right\}$. The distance set is obtained between each testing sample $W_{ls}$ and training samples $T_{ts}$. We can select the first *K* points in ascending order, and the sample label $W_{ls}$ the with highest frequency is considered the category of the testing sample. Euclidean distance (*L*^2^-norm) is adopted as a similarity criterion, where the distance between the testing sample and training sample is shown in Equation (22):

(22) $$d_{\left(\mathrm{ts},\mathrm{ls}\right)}=\sqrt{{\left(T_{ts}-W_{ls}\right)}^{2}}$$

where *T_ts_* is the *ts*th training sample (*ts* = 1, 2, …, *TS*), $W_{ls}$ is the *ls*th testing sample (*ls* = 1, 2, …, *SS*).

### 4.5. Outline of the Proposed Method

**Figure 12:**
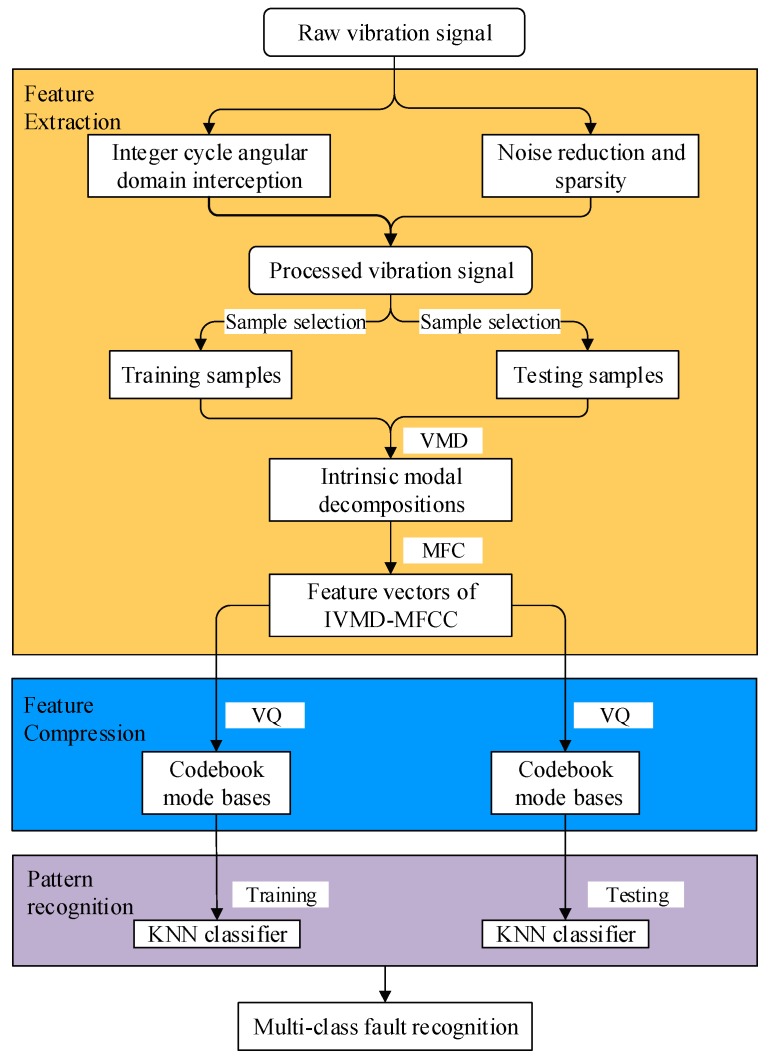
Schematic of the proposed method. KNN, K-nearest neighbor; VQ, Vector quantization.

As mentioned in the above sections, a comprehensive fault detection and diagnosis method for a diesel engine under multiple operation conditions is proposed, based on IVMD-MFCC-VQ-KNN, where IVMD-MFCC is allocated to feature extraction, Vector quantization (VQ) is allocated to dimension reduction, and KNN is allocated to fault identification. The availability of the proposed approach is examined by diesel engine valve clearance fault experiments under different conditions. The flow diagram containing the main implementation steps is described in Figure 12.

## 5. Diagnosis Results of Valve Clearance Fault

### 5.1. Results Analysis of the Proposed Method

Considering some actual engineering occasions, in this work, the proposed methods are validated with two datasets of diesel engine valve faults. However, in one of the datasets, the vibration signals are purely experimental signals, in which the noise level is relatively low. However, in the other dataset, the noise level of the vibration signal is strengthened by the strong noise with an extremely low signal-to-noise ratio (SNR = −5 dB). The datasets and other parameters remained invariable when the experiment of each valve state was carried out repeatedly six times on two datasets. The confusion matrix, as is common in studies, is used to evaluate the classifier effect in this paper, where the columns represent the predicted class and the rows plot the actual class of the testing samples. The performance of the proposed method is evaluated in six time experiments using three indicators of the confusion matrix, including accuracy (AC), precision (PR), and sensitivity (SE). AC is used to evaluate the classification of the performance of the overall model, and PR detects whether a testing sample is normal or faulty, while SE can measure the sensitivity level of a certain state.

The first trial detailed results for seven valve clearance states with low-level noise and high-level noise, as listed in Table 3 and Table 4, respectively. It can be seen from Table 3 that, for the inlet valve faults occurring separately, some inaccurate results are gained for SFI and LFI due to being mistaken for NVC, with lower PR values of 97.76% and 95.71%, respectively, whereas the PR in the SFE, SFIE, and LFIE classes is equal to 100%. This shows that the probability of correctly detecting the exhaust valve faults from vibration signals is higher than the correct detection of intake valve faults. Similarity, in Table 4, the PR values in SFI and LFI are 97.14% and 94.70%, whereas the PR values related to exhaust valve faults are higher than 98%. All these also prove that the probability of recognizing exhaust valve fault signals is higher compared to the intake valve fault signals with strong noise. It can be also observed that, out of 980 normal clearance samples, approximately 2.45% of the signals are wrongly classified as fault signals. Likewise, for the SFI, LFI, and LFE faults, a total of 6.93% of fault clearance samples were mistaken as normal clearance samples. Similarity, in Table 4, approximately 4.08% of the signals are wrongly classified as faults and 10.4% are wrongly recognized as fault signals. Furthermore, both with the low-level noise and with the high-level noise, the results indicate excellent performance, in light of sensitivity, with a high SE value, which exceeds 90%, except for NVC, whose lowest value is 87.52%.

The overall performance of the proposed method is also described in Table 3 and Table 4. The higher overall accuracy, average precision, and sensitivity of 98.66%, 98.66%, and 98.70% were obtained using experimental signals with a low-level noise. Furthermore, the overall accuracy, average precision, and sensitivity (97.46%, 97.47% and 96.91%) of the proposed method slightly reduce under the influence of strong noise.

In conclusion, strong noise has little influence on the performance of the proposed method. In order to verify the robustness to the number of samples, the proportion of each state samples of dataset without noise was predefined to five levels: 60/1040, 120/980, 180/920, 240/860, 300/800. Table 5 exhibits the effects of training and testing the sample proportion on the performance of the proposed method. It can be observed from Table 5 that the proposed method exhibits good performance for all training/testing samples in terms of average accuracy, precision, and sensitivity. The average accuracy and precision is higher with more training samples, of which the average accuracy of 99.72% with a proportion of 240/860 and the average precision of 99.15% with a proportion of 300/800 were highest. The major reason is that a greater number of training samples means ore fault information, which contributes to the feature learning of vibration signals. However, the average calculation time increases with the number of training samples. Thus, it is also a great challenge to define the number of training/testing samples for improving the overall performance of the diesel engine fault diagnosis system. As can be seen from Table 5, 120/980 of the training/testing samples show the best proportion according to four synthetically appraisable factors, which not only ensure accuracy, precision, and sensitivity, but also save calculation time.

### 5.2. Results Analysis of Comparative Methods

**Figure 13:**
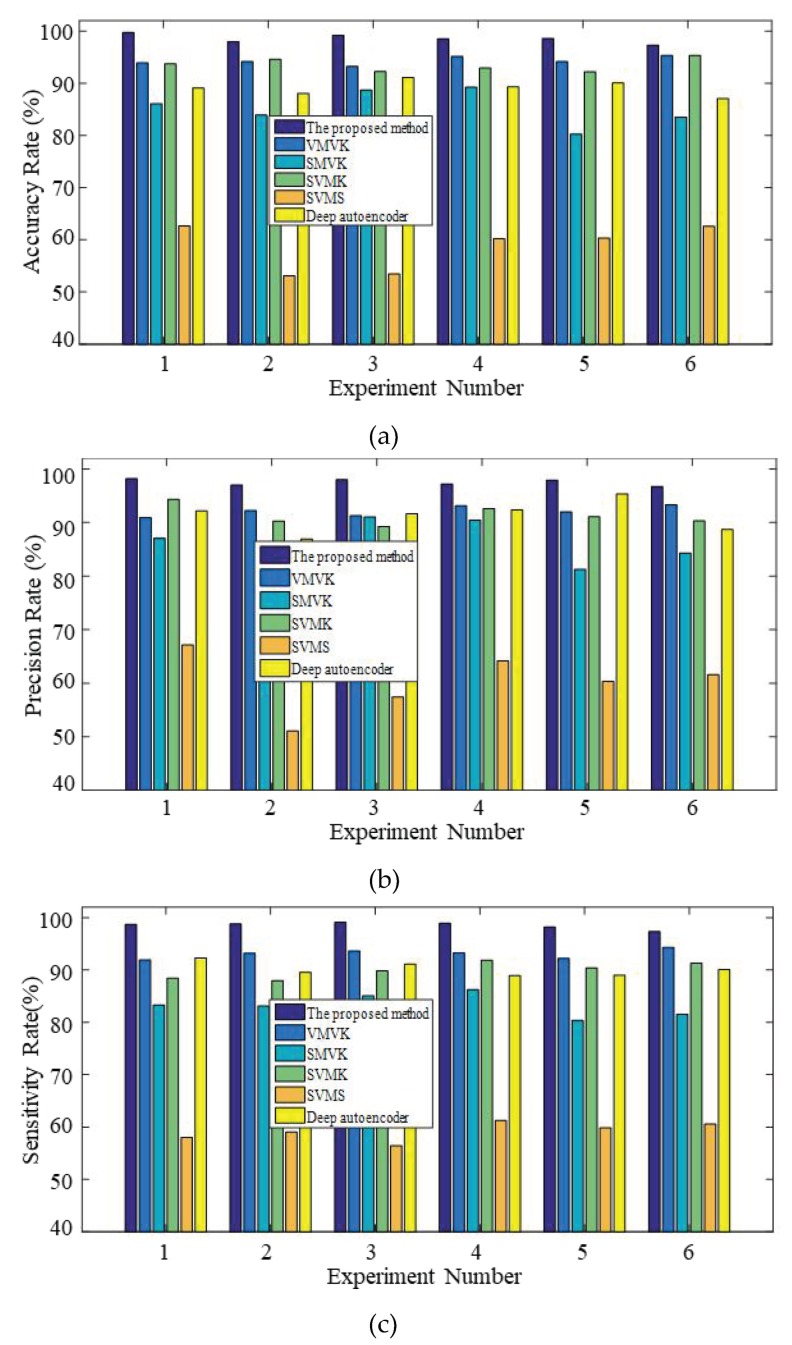
The single diagnosis results of the contrastive methods for the six experiments: (**a**) AC; (**b**) PR; (**c**) SE.

To further prove the effectiveness of the proposed method, the process of fault diagnosis for valve clearance are automatically accomplished by using a deep autoencoder with no human intervention such as signal preprocessing, manual feature extraction, and feature dimension reduction. In this paper, the architecture of the deep autoencoder is set to five layers, including the input layer, three hidden layer, and output layer, of which the number of units are 1024, 500, 260, 60, and 7, respectively. The hyper-parameters are initially selected by a few general principles and finally tested by the experimental dataset, of which learning rate, sparsity parameter, momentum, and weight of the sparsity penalty terms and weight decay are 0.02, 0.01, 0.08, 0.017, and 4.736 [38,39]. This method consists entirely of five steps including signal improvement (SI), variational mode decomposition (VMD), Mel frequency cepstrum coefficient (MFCC), and K-Nearest Neighbor (KNN), which is abbreviated as IVMD-MFCC-VQ-KNN. Meanwhile, four comparative methods are also presented to demonstrate the importance of each step. The first comparative method is the VMD-MFCC-VQ-KNN without the process of signal improvement (VMVK). The second comparative method is the SI-MFCC-VQ-KNN without the process of VMD (SMVK). The third comparative method is the SI-VMD-MFCC-KNN without the process of vector quantization (SVMK) and the fourth comparative method is the SI-VMD-MFCC-VQ-SVM replacing KNN classifiers with SVM classifiers (SVMS). The kernel function of SVM is RBF, and the penalty index and radius of RBF are set to 20 and 0.081, respectively [40]. The single diagnosis results of the contrastive methods depicted in Figure 13 and Table 6 exhibits the average accuracy, average precision, average sensitivity, and calculation time of six experiments. The single diagnosis results in Figure 13 demonstrate that the proposed method can detect the valve clearance faults effectively, yielding good performance for AC, PR, and SE, for which the values are more than 95% for each testing experiment. From the average diagnosis results of the contrastive methods in Table 6, it is evident that the average values of AC, PR, and SE are 98.54%, 97.50%, and 98.50%, respectively, which were calculated by the proposed method, which yielded higher results than other methods. For example, the average values of AC, PR, and SE of VMVK and SMVK are slightly lower than the proposed method, due to the absence of the signal improvement and VMD, although the average calculation time is reduced. Furthermore, the average calculation time of the proposed method is 55.30 s, and the processing speed is increased significantly compared to the SVMK method, of which the average calculation time is 80.20 s. It is noteworthy that the calculation time of each experiment is always less than 60 s through the proposed method. The results also show that due to the insufficient training data and calculation complexity, the deep autoencoder does not perform as well as the proposed method.

## 6. Conclusions

In this study, a novel detection and diagnosis approach is investigated to diagnose diesel engine faults under multiple operation conditions. Introducing an improved Mel frequency transformation and adaptive correlation threshold processing into the VMD and MFCC framework, we proposed a representative feature called IVMD-MFCC under multiple operation conditions. The vector quantization plays a vital role in dimension reduction for removing redundant information and improving model performance. The K-Nearest Neighbor is used as the classifier. The vibration signal collected from diesel engine cylinder head is utilized to demonstrate the availability of proposed approach under the seven valve clearance states and twelve operating conditions. In light of the experimental results, it will undoubtedly be concluded that the proposed approach gives good overall performance in the light of AC, PR, and SE. In particular, it is no doubt proven that this will be a good start for constructing a vibration signal fault feature similar to the “acoustical system” based on the Mel scale principle.

## Figures and Tables

**Table 1:** Description of the valve state.

Case	Intake Valve	Exhaust Valve	Number of Training Samples	Number of Testing Samples
Normal valve clearance (NVC)	0.3	0.5	120	980
Small clearance fault of intake valve (SFI)	0.25	0.5	120	980
Large clearance fault of intake valve (LFI)	0.4	0.5	120	980
Small clearance fault of exhaust valve (SFE)	0.3	0.45	120	980
Large clearance fault of exhaust valve (LFE)	0.3	0.6	120	980
Small clearance fault of intake and exhaust valve (SFIE)	0.25	0.45	120	980
Large clearance fault of intake and exhaust valve (LFIE)	0.4	0.6	120	980

**Table 2:** Twelve experiment operating conditions.

Number	Speed (rpm)	Load (N·m)	Number	Speed (rpm)	Load (N·m)
1	1500	700	7	1800	1600
2	1500	1000	8	2100	700
3	1500	1300	9	2100	1000
4	1800	700	10	2100	1300
5	1800	1000	11	2100	1600
6	1800	1300	12	2100	2200

**Table 3:** Confusion matrix of seven valve clearance states of first trial with the low-level noise.

		Predicted Class									
		NVC	SFI	LFI	SFE	LFE	SFIE	LFIE	AC (%)	PR (%)	SE (%)
**Actual Class**	**NVC**	956	4	8	3	2	5	2		97.55	93.36
	**SFI**	22	958	0	0	0	0	0		97.76	99.58
	**LFI**	42	0	938	0	0	0	0		95.71	99.15
	**SFE**	0	0	0	980	0	0	0		100	99.69
	**LFE**	4	0	0	0	976	0	0		99.59	99.80
	**SFIE**	0	0	0	0	0	980	0		100	99.49
	**LFIE**	0	0	0	0	0	0	980		100	99.80
**Overall Performance**									98.66	98.66	98.70

**Table 4:** Confusion matrix of seven valve clearance states of first trial with the high-level noise.

		Predicted Class									
		NVC	SFI	LFI	SFE	LFE	SFIE	LFIE	AC (%)	PR (%)	SE (%)
**Actual Class**	**NVC**	940	5	11	5	3	7	9		95.92	87.52
	**SFI**	28	952	0	0	0	0	0		97.14	94.48
	**LFI**	52	0	928	0	0	0	0		94.70	98.83
	**SFE**	12	0	0	968	0	0	0		98.78	99.49
	**LFE**	10	0	0	0	970	0	0		98.98	99.69
	**SFIE**	17	0	0	0	0	963	0		98.27	99.28
	**LFIE**	15	0	0	0	0	0	965		98.47	99.08
**Overall Performance**									97.46	97.47	96.91

**Table 5:** The effects of the sample proportion on the performance of the proposed method.

Number of Training/Testing Samples	Average Accuracy (%)	Average Precision (%)	Average Sensitivity (%)	Average Calculation Time (s)
60/1040	95.71	93.18	96.12	52.32
**120/980**	**98.54**	**97.50**	**98.50**	**55.30**
180/920	98.86	97.66	98.17	65.15
240/860	99.72	98.80	98.35	80.38
300/800	99.70	99.15	98.30	100.86

**Table 6:** The average diagnosis results of the contrastive methods for the six experiments.

Methods	Average Accuracy (%)	Average Precision (%)	Average Sensitivity (%)	Average Calculation Time (s)
The proposed method	98.54	97.50	98.50	55.30
VMVK	94.31	92.15	93.08	50.15
SMVK	85.27	86.56	83.25	40.76
SVMK	93.50	91.31	89.95	80.20
SVMS	58.72	60.30	59.22	42.38
Deep autoencoder	89.11	91.20	90.15	70.86

## Abbreviations

**Abbreviations**		*ss*	The *ss*th label of testing sample
AC	Accuracy	*Mel*	Mel frequency
EEMD	Ensemble empirical mode decomposition	*f*	Hertz frequency
EVCI	Exhaust valve closing impact	*Y*	Fast Fourier transform of mode component $u_{k}^{l}(s)$
FFT	Fast fourier transform	*t*	Time
FI	Fire impact	*E*	The linear spectrum energy of *p*th line of each frame signal
FINC	Fire impact adjacent cylinder	$u_{k}$	The *k*th decomposed mode components
IVCI	Intake valve closing impact	$\omega_{k}$	Central frequency
IVMD-MFCC	Improved vibrational mode decomposition and Mel frequency cepstrum coefficient	${\overset{⌢}{u}}_{k}^{n+1}(\omega )$, $\omega_{k}^{n+1}$, ${\overset{⌢}{\lambda }}^{n+1}(\omega )$	The corresponding Fourier transformation
LBG	Linde -Buzo-Gray	*x*(*t*)	Original signal
LDA	Linear discriminative analysis	*j*	Imaginary unit
LFE	Large clearance fault of exhaust valve	*p*	The *p*th line in frequency-domain
LFI	Large clearance fault of intake valve	*N*	The data point number of $u_{k}^{l}(s)$
LFIE	Large clearance fault of intake and exhaust valve	s	The *s*th data of $u_{k}^{l}(s)$
LMD	Local mean decomposition	*L*	the integer cycle signal length
Ma Tec	Maintenance technicians	*M*	Number of sliding windows
MFC	Mel frequency cepstrum coefficient	IM	IVMD-MFCC feature
MFCC	Mel frequency cepstrum coefficient	*l*	the *l*th sub-frame signal
NVC	Normal valve clearance	*R_rms_*	Mean square root of correlation coefficient
PCA	Principal component analysis	*d*	Euclidean distance between X and Y*_c_*
PR	Precision	*n*	Number of iterations
IMFs	Intrinsic mode functions	*J*	The number of feature vector subspaces
ISOMap	Isometric feature mapping	*ρ*	A constant
SE	Sensitivity	*F*	The total number of Mel filter banks
SHM	Structural health monitoring	*fr*	The *fr*th Mel filter bank
SI	Signal improvement	X	Training set of vector quantization
SFE	Small clearance fault of exhaust valve	Y_*c*_	Codebook of *c*th subspace
SFI	Small clearance fault of intake valve	S	Mel-frequency spectrum energy of each frame signal
SFIE	Small clearance fault of intake and exhaust valve	*SU*	Number of training set subspaces
SMVK	The proposed method without VMD	*T_ts_*	Training sample of KNN
SVMK	The proposed method without vector quantization	*W_ls_*	Label set of *T_n_*
SVMS	The proposed method replacing KNN with SVM	*S_SS_*	Testing sample of KNN
STFT	Short time Fourier transform spectrum	*N_wl_*	Length of sliding window
VMD	Variational mode decomposition	*N_ss_*	Moving step size of sliding window
VMVK	The proposed method without signal improvement	*m*	The *m*th data point of the sliding window
VQ	Vector quantization	Convergence criteria of vector quantization	The *m*th data point of the sliding window
**Symbols**		Ψ	Total distortion of all subspaces
γ	Convergence criteria	*Ra*	Correlation coefficient between (*a* − 1)th and *a*th sliding window
α	A castigatory quadratic	*TS*	Number of training sample
λ	Lagrangian multiplicator operator	*SS*	Number of testing sample
ℒ	extended Lagrangian function	*ts*	The *ts*th training sample
δ	Dirac distribution function	*ls*	The *ls*th label of training sample
*τ*	Noise tolerance parameter		
